# Epigenetic Alterations in Prenatal Stress Mice as an Endophenotype Model for Schizophrenia: Role of Metabotropic Glutamate 2/3 Receptors

**DOI:** 10.3389/fnmol.2018.00423

**Published:** 2018-11-30

**Authors:** Francesco Matrisciano, Erbo Dong, Ferdinando Nicoletti, Alessandro Guidotti

**Affiliations:** ^1^Department of Psychiatry, Psychiatric Institute, College of Medicine, University of Illinois Chicago, Chicago, IL, United States; ^2^Department of Psychiatry, Center for Alcohol Research in Epigenetics College of Medicine, University of Illinois Chicago, Chicago, IL, United States; ^3^Department of Physiology and Pharmacology, University of Rome “Sapienza”, Rome, Italy; ^4^IRCCS, Neuromed, Pozzilli, Italy

**Keywords:** mGlu2/3 receptors, schizophrenia, clozapine, epigenetics, prenatal stress

## Abstract

Mice subjected to prenatal restraint stress (*PRS* mice) showed biochemical and behavioral abnormalities consistent with a schizophrenia-like phenotype (Matrisciano et al., 2016). PRS mice are characterized by increased DNA-methyltransferase 1 (DNMT1) and ten-eleven methylcytosine dioxygenase 1 (TET1) expression levels and exhibit an enrichment of 5-methylcytosine (5MC) and 5-hydroxymethylcytosine (5HMC) at neocortical GABAergic and glutamatergic gene promoters. Activation of group II metabotropic glutamate receptors (mGlu2 and−3 receptors) showed a potential epigenetically-induced *antipsychotic* activity by reversing the molecular and behavioral changes observed in PRS mice. This effect was most likely caused by the increase in the expression of growth arrest and DNA damage 45-β (Gadd45-β) protein, a molecular player of DNA demethylation, induced by the activation of mGlu2/3 receptors. This effect was mimicked by clozapine and valproate but not by haloperidol. Treatment with the selective mGlu2/3 receptors agonist LY379268 also increased the amount of Gadd45-β bound to specific promoter regions of reelin, BDNF, and GAD67. A meta-analysis of several clinical trials showed that treatment with an orthosteric mGlu2/3 receptor agonist improved both positive and negative symptoms of schizophrenia, but only in patients who were early-in-disease and had not been treated with atypical antipsychotic drugs (Kinon et al., 2015). Our findings show that PRS mice are valuable model for the study of epigenetic mechanisms involved in the pathogenesis of schizophrenia and support the hypothesis that pharmacological modulation of mGlu2/3 receptors could impact the early phase of schizophrenia and related neurodevelopmental disorders by regulating epigenetic processes that lie at the core of the disorders.

## Highlights

Prenatal restraint stress (*PRS*) in mice showed epigenetic changes and behavioral abnormalities consistent with a schizophrenia-like phenotype.
Prenatal stress (PRS) represents a suitable non-pharmacological model to study schizophrenia and to develop novel antipsychotics.
Activation of mGlu2/3 receptors corrects the altered epigenetic and behavioral changes induced by prenatal stress in mice.
Both clozapine and the mGlu2/3 receptor agonist LY379268 acted as epigenetic agents targeting specifically DNA methylation reversing the molecular and behavioral alterations in PRS mice.

## Introduction

**Figure 1:**
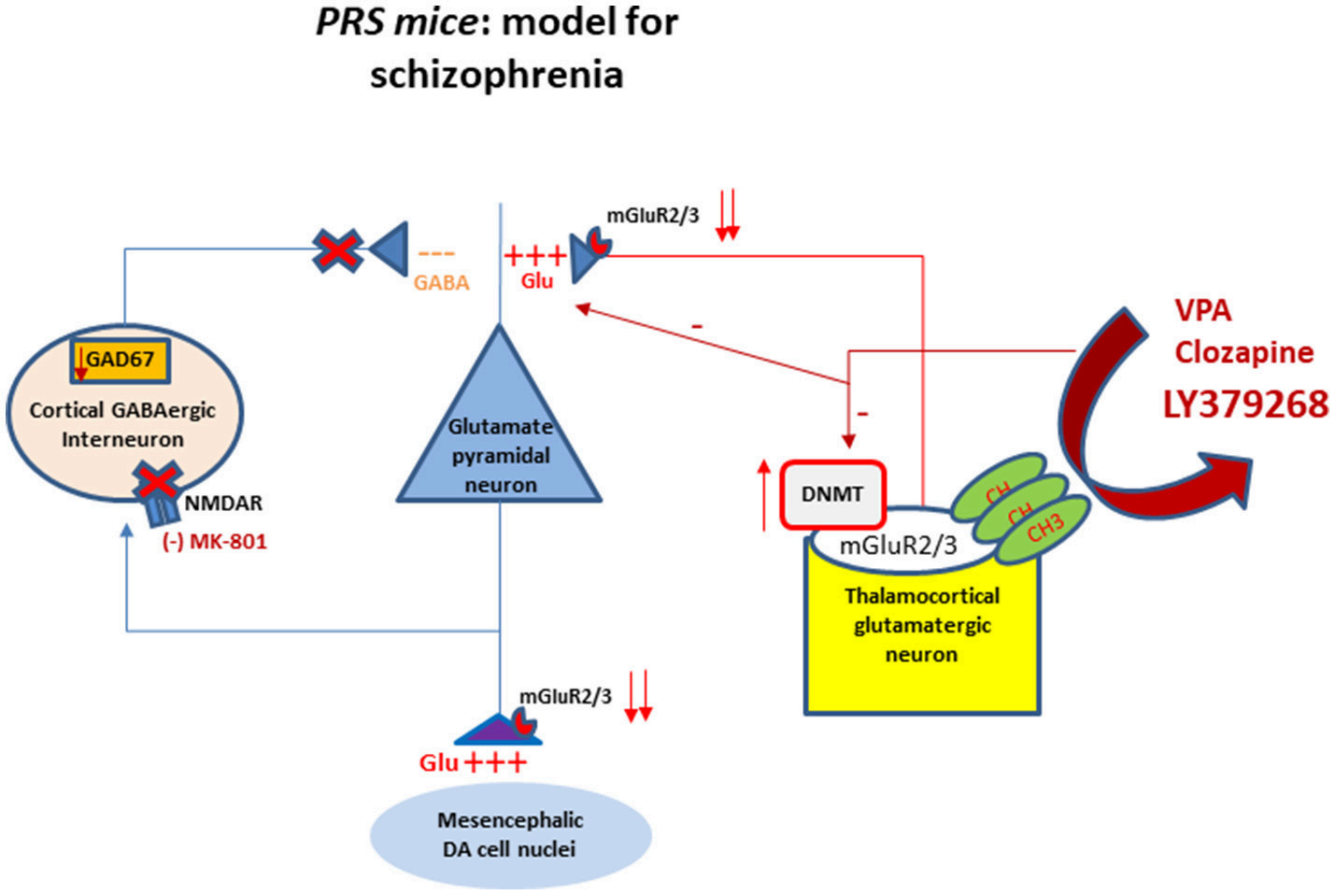
Schematic representation of the interactions between GABAergic and glutamatergic neurotransmission in cortical-limbic structures of PRS mice. The cartoon shows altered DNA promoter hypermethylation (increase in DNMT) occurring at the mGlu2/3 receptors gene promoter and their decreased expression at presynaptic level of thalamocortical glutamatergic neurons. The downregulation of mGlu2/3 receptors at the axon terminal of thalamocortical glutamatergic neurons results in the hyperactivation of glutamatergic pyramidal neurons. This activation is facilitated by a decrease of GABAergic feedback inhibition on pyramidal neurons. The hypofunction of GABAergic interneurons is mediated by a downregulation of NMDA receptor function as suggested by the behavioral hypersensitivity to small doses of NMDA receptor blocker MK-801 (Matrisciano et al., 2013). The same fibers project to subcortical areas causing an excessive firing and dopamine release. The cartoon shows also the mGlu2/3 receptors at presynaptic level of the thalamocortical fibers as potential target for pharmacological interventions such as the mGlu2/3 receptor agonist LY379268, valproate and clozapine to restore the normal balance between GABA and glutamate through epigenetic mechanisms. DA, dopaminergic; DNMT, DNA methytransferase; NMDA, N-methyl-D-aspartate; CH3, methyl group.

Schizophrenia is a major psychotic disorder which affects one percent of the world's population and usually leads to a severe mental disability (Ribe et al., 2015). All marketed antipsychotic drugs antagonize D2 dopamine and 5-HT_2A_ serotonin receptors, showing good clinical efficacy in improving positive symptoms, and moderate activity in improving negative symptoms of schizophrenia. None of these drugs has significant activity on cognitive symptoms associated with schizophrenia, with the possible exception of clozapine (Lieberman et al., 2005). Treatment of cognitive and negative symptoms remains an unmet need in the treatment of schizophrenia, and this encourages the identification and validation of novel drug targets. Etiology of schizophrenia is still unknown despite the recent progresses made possible by molecular genetics and functional neuroimaging. It is generally believed that schizophrenia is not caused by a single factor, but results from the convergence of genetics and environmental factors. Recently, an imbalance between GABA and glutamate neurotransmission has been suggested as a key mechanism underlying the pathophysiology of schizophrenia. Drugs that block the activity of NMDA receptors present on cortical-limbic GABAergic interneurons, such as ketamine or phencyclidine (PCP) are capable to replicate the full range of psychotic symptoms, including hallucinations (Kristiansen et al., 2007; Meltzer et al., 2011). Thus, novel antipsychotic drug development should focus on the GABA and glutamate systems, which act upstream of the dopamine circuit, and are primarily involved in the pathophysiology of the disorder (Figure 1). A hypofunction of the NMDA receptors on GABAergic interneurons, particularly fast-spiking, parvalbumin-positive chandelier and basket cells, leads to an overactivity of pyramidal neurons and to an impairment of network oscillations that underlie multiple domains of cognitive function (Homayoun and Moghaddam, 2007; Gonzalez-Burgos and Lewis, 2008). The release of GABA is crucial for the normal firing of pyramidal neurons in the prefrontal cortex and for the equilibrium of the subcortical regions fundamental for optimizing cognitive and emotional function (Benes et al., 2007). Schizophrenia is a chronic devastating disorder that leads to a severe disability at relatively young age. It has a peculiar pathological course starting with the prodromal phase followed by a first episode, which occurs around adolescence or young adult age (Millan, 2012). Evidence suggests that epigenetic changes, occurring during early development as a result of the combination of a predisposing genetic background, in shaping the premorbid phase of the disease, and environmental factors, acting as “second hits,” precipitate the onset of schizophrenia (Guidotti et al., 2005). We and others have studied the epigenetic hallmarks of schizophrenia in postmortem human brain tissue. Moving from these findings, we have investigated whether the same epigenetic changes occur in the brain of mice subjected to prenatal stress at different stages of postnatal development. The purpose of this review is to provide an update (i) of our current findings and knowledge of the topic of *neuroepigenetics* in schizophrenia, (ii) of the role of metabotropic glutamate 2/3 receptors in prenatally stressed mice (PRS mice) as potential targets for novel antipsychotics; and (iii) to show our more recent observations on the epigenetic effects induced by the mGlu2/3 receptors agonist, LY379268, and by clozapine.

## *Epigenetic* Changes in Schizophrenia

Neuroepigenetic dysregulations were detected in the hippocampus and cortex of brain of patients affected by schizophrenia (Numata et al., 2014; Dong et al., 2015). *Epigenetics* is defined as modifications of the genome, heritable during cell division, that do not involve a change in DNA sequence. Epigenetic mechanisms are considered to mediate gene-environment interplay during the entire lifespan. Several clinical evidence support a role of altered epigenetic mechanisms underlying embryonic, postnatal, and adult neurogenesis (Roth et al., 2011). Aberrations in the epigenetic regulation machinery have been hypothesized in neurodevelopmental disorders, such as schizophrenia and autism spectrum disorders (Zhubi et al., 2017). A growing body of evidence from Dr. Guidotti's group (Matrisciano et al., 2012, 2013, 2016) and other researchers (Meaney and Szyf, 2005; Benes et al., 2007; McGowan and Szyf, 2010) suggest that epigenetic modifications of DNA (promoter methylation) and chromatin remodeling induced by environmental factors, including stress, may contribute to the complex phenotypes of neuropsychiatric disorders, such as schizophrenia. DNA methyltransferases (DNMT1 and 3a) (the enzymes that transfer a methyl group from S-adenosylmethionine to carbon 5 of the cytosine pyrimidine ring embedded in cytosine-phospho-guanine [CpG] islands containing promoters), and ten-eleven translocation hydroxylase (TET 1,2,3), (the enzymes that catalyze the conversion of 5MC to 5HydroxyMC), are important components of the DNA- methylation/demethylation pathways regulating the expression of key molecules involved in brain development and maturation. Importantly, the prefrontal cortex GABAergic interneurons of schizophrenia patients express an increase in DNMT1 and 3a, and an increase in TET1 associated with deficits in GABAergic function (Guidotti et al., 2011). This includes the downregulation of the glutamic acid decarboxylase 67 (GAD), reelin, GABA reuptake transporters and brain derived neurotrophic factor (BDNF), which are essential for neurogenesis, neurodevelopmental neuronal migration and synaptic plasticity. In addition to alterations of GABAergic transmission (Veldic et al, 2007; Lewis and González-Burgos, 2008; Kundakovic et al., 2009; Marek, 2010; Niswender and Conn, 2010; Marín, 2012), there are evidence of altered glutamatergic transmission involving both ionotropic glutamate receptors such as NMDA receptors present post-synaptically on GABAergic interneurons (Figure 1) and metabotropic glutamate receptors such as mGlu2/3 receptors located at least in part pre-synaptically on thalamocortical glutamate axon terminals [Benes et al., 2007; Stansley and Conn, 2018; Figure 1] where they modulate the glutamatergic transmission by restoring the hyperactivity of the cortical pyramidal neurons (Figure 1). Clinically, while NMDA receptor agonists are not fully considered as antipsychotic drugs due to their potential excitotoxicity effects and neuronal damage, metabotropic glutamate 2/3 receptors represent a suitable target for glutamatergic tone regulation.

Taken together, these observations suggest that the epigenetic hypothesis of schizophrenia is supported by the following evidence obtained in postmortem human brain: (i) alterations of glutamic acid decarboxylase 67 (gene symbol = GAD1), DNMT and reelin in GABAergic neurons, and (ii) of brain derived nerve growth factor (BDNF), vesicular glutamate transporter (VGLUT1) and (iii) alterations in mGlu2/3 receptors in glutamatergic neurons (Guidotti et al., 2011; Nicoletti et al., 2011).

## The *PRS* as a Suitable Animal Model to Study Neurodevelopmental Disorders

Prenatal or early-life stress, through changes in the epigenetic mechanisms, has been considered a predisposing factor for major neuropsychiatric disorders including schizophrenia, bipolar disorders, and autism spectrum disorder. Time- and spatial-dependent neurodevelopmental cues associated with neuronal differentiation and synaptic plasticity support the hypothesis that these disorders might originate even before birth. Interestingly, we have reported that adult offspring of mice exposed to repeated episodes of restraint stress during pregnancy, named *PRS mice*, exhibit a schizophrenia-like behavioral phenotype characterized by hyperactivity, stereotyped and compulsive behavior, deficits in social interaction and pre-pulse inhibition (PPI), altered fear conditioning, object recognition, and hypersensitivity to N-methyl D-aspartate (NMDA) receptor blockers (Matrisciano et al., 2016). This behavioral phenotype recapitulates positive and negative symptoms, as well as cognitive dysfunction displayed by patients affected by schizophrenia (Matrisciano et al., 2013). PRS mice also show a deficit in cortical GABAergic innervation, which is expected to cause abnormal synchronization of the firing rate of pyramidal neurons, a putative electrophysiological substrate of cognitive dysfunction in psychotic patients and neurodevelopmental animal models of SZ (Gonzalez-Burgos and Lewis, 2008). In addition to alterations of GABAergic system, PRS mice show molecular disruption in chromatin remodeling at genes expressed in glutamatergic neurons, such mGlu2/3 receptors (Figure 1). These molecular changes in PRS mice are similar to those observed in the brain of schizophrenia patients, suggesting a strong correlation between the aberrant epigenetic GABAergic/glutamatergic mechanisms and psychotic symptoms. Table 1 summarize the behavioral and molecular features observed in *PRS mice* and *Schizophrenia patients*.

**Table 1:** Epigenetic and behavioral deficits found in schizophrenia patients and in PRS mice.

**Schizophrenia**	**PRS mice**
+ Positive (stereotype behaviors)	+ stereotype behaviors
+ Negative (SI deficits) symptoms	+ SI deficits symptoms
+ sensitivity to NMDAR blockers	+ sensitivity to NMDAR blockers
+ cognitive symptoms	+ information processing deficit (PPI, fear conditioning)
Reduction of the GAD67, RELN, BDNF expression levels	Reduction of the GAD67, RELN, BDNF expression levels in frontal cortex
Increase of DNMT1, 3A and TET1 expression levels	Increase of DNMT1, 3A and TET1 expression levels in frontal cortex
Increase in 5MC and 5HMC enrichment at Gadl, Reln and Bdnf promoters	Increase in 5MC and 5HMC enrichment at Gadl, Reln and Bdnf promoters

(+): presence. Abbreviations: BDNF, brain-derived neurotrophic factor; DNMT1, DNA-methyltransferase 1; TET1, ten-eleven methylcytosine dioxygenase 1; SI, social interaction, NMDA, N-methyl D-aspartate; PPI, pre-pulse inhibition; GAD67, glutamic acid decarboxylate; 5HMC, 5-hydroxymethylcytosine; 5MC, 5methylcytosine

## Role of Metabotropic Glutamate 2/3 Receptors in Schizophrenia

Disruption in the glutamatergic system is considered to play a key role in the pathophysiology of schizophrenia (Akbarian et al., 1995). It has been reported that *patients* affected by schizophrenia respond only partially to standard “monoaminergic” antipsychotic drugs (Lieberman et al., 2008; Meltzer, 2013). The lack of a full recovery from negative and cognitive symptoms gave the impetus to investigate different molecular targets including mGlu2 and mGlu3 receptors. mGlu receptors, which belong to class C of the G protein-coupled receptors form a family of eight subtypes traditionally subdivided into three groups based on sequence homology, intracellular signaling and pharmacological profile. mGlu1 and mGlu5 receptors (group I) are coupled to Gq/11 and their activation stimulates polyphosphoinositide hydrolysis with ensuing formation of inositol-1,4,5-trisphosphate and diacylglycerol. These receptors are localized in the peripheral portions of postsynaptic densities [reviewed by (Nicoletti et al., 2011)]. mGlu2 and mGlu3 receptors are coupled to Gi/o and their activation inhibits adenylyl cyclase activity and modulate the activity of calcium and potassium channels. Both receptors are localized in axon terminals where they negatively modulate neurotransmitter release [(Nicoletti et al., 2011); Figure 1]. However, recent findings indicate that mGlu3 receptors are also localized in postsynaptic densities, where they boost mGlu5 receptor signaling (Di Menna et al., 2018). mGlu4, mGlu7, and mGlu8 receptors (group III) are also coupled to Gi/o and are found in presynaptic terminals close to the active zone of neurotransmitter release (Nicoletti et al., 2011). Symptoms of schizophrenia are thought to be associated, at least in part, with hyperactive and dysregulated glutamatergic neurotransmission in key brain regions, such as the thalamus, prefrontal cortex, and limbic system. Based on this evidence, pharmacological activation of mGlu2/3 receptors may ameliorate the schizophrenia symptoms through a decrease in glutamate release thereby reducing synaptic firing due to the particular synaptic distribution of these receptors and causing neuroprotective effects. Interestingly, PRS mice showed a decrease in the expression of mGlu2 and mGlu3 receptor mRNA and proteins in the frontal cortex. This decrease manifest at birth and, at least for mGlu2 receptors, persisted in adult life associated with an increase in the DNMT binding to the gene promoter [for more details see (Matrisciano et al., 2013)] suggesting an epigenetic regulation of the receptors induced by prenatal stress and it may reflect a key factor for the pathogenesis of the disease.

In schizophrenia research, particular attention has been paid to group-II mGlu receptors on the basis of genetic and pharmacological data (Gregory and Conn, 2015).

An initial hypothesis was that activation of mGlu2/3 receptors could improve psychotic symptoms by inhibiting glutamate release (Battaglia et al., 1997), and, therefore, restraining the hyperactivity of pyramidal neurons associated with schizophrenia. However, this mechanism may also amplify the defect in glutamate-mediated activation of GABAergic interneurons, thus worsening the “glutamatergic hypofunction” that underlies cognitive dysfunction in schizophrenia. The evidence that activation of mGlu2 receptors inhibits electrophysiological responses mediated by 5-HT_2A_ receptors at thalamo-cortical synapses (Aghajanian and Marek, 2000) shed new light into the defensive role played by mGlu2 receptors in schizophrenia. Javier Gonzales-Maeso and his Associates have consistently shown that mGlu2 and 5-HT_2A_ receptors form functional multimeric complexes, in which mGlu2 receptors negatively modulate 5-HT_2A_ receptor signaling (González-Maeso et al., 2008). Interestingly, opposite changes in the expression of mGlu2 and 5-HT_2A_ receptors were found in postmortem brain tissue from patients affected by schizophrenia, with the physiological balance between the two receptors being shifted toward 5-HT_2A_ receptors (Muguruza et al., 2013). This is nicely consistent with the reduced expression of mGlu2 receptors in the prefrontal cortex found across the postnatal development of PRS mice, which show a schizophrenia-like phenotype in the adult life (Matrisciano et al., 2016). Interestingly, treatment with atypical antipsychotics down-regulates mGlu2 receptors in the prefrontal cortex as a result of an epigenetic mechanism that, in this particular case, is mediated by histone deacetylation at the *Grm2* gene promoter (Kurita et al., 2012). This contributes to explain why schizophrenic patients who had received a previous treatment with atypical antipsychotics failed to respond to pomeglumetad (Kinon et al., 2015), and raise the interesting possibility that acetylating drugs (e.g., inhibitors of histone deacetylases) may boost the activity of mGlu2 receptor agonists or positive allosteric modulators (PAMs) in the clinic. The mGlu2-centric scenario in the treatment of schizophrenia is supported by a large number of preclinical studies showing that the behavioral effects of orthosteric mGlu2/3 receptor agonists in behavioral tests that are predictive of antipsychotic activity are abrogated in mGlu2 receptor knockout mice, and that selective mGlu2 receptor PAMs display robust antipsychotic-like activity in rodents (Gerwirtz and Marek, 2000; Schoepp and Marek, 2002; Egan et al., 2004; Benneyworth et al., 2007; Patil et al., 2007; Conn et al., 2008). However, the following observations bring to a re-evaluation of mGlu3 receptors in schizophrenia research: (i) mice lacking mGlu3 receptors display a psychotic-like phenotype (Lainiola et al., 2014), and show developmental abnormalities in cortical GABAergic transmission (M. Cannella et al., unpublished observations); (ii) polymorphic variants of GRM3 are consistently associated with schizophrenia [reviewed by Maj et al., 2016], whereas no variants of GRM2 have been associated with psychiatric disorders; and, (iii) as reported above, mGlu3 receptors boost mGlu5 receptor signaling (Di Menna et al., 2018), and mGlu5 receptors are candidate drug targets in the treatment of schizophrenia [reviewed by Foster and Conn, 2017]. It is noteworthy that expression of mGlu3 receptors was also reduced in the prefrontal cortex of PRS mice, although this reduction was significant at 1 and 9 days of postnatal life, but not after weaning (Matrisciano et al., 2013). In clinical studies, systemic treatment with pomeglumetad methionyl, an oral prodrug of the mGlu2/3 receptor agonist, LY404039, showed antipsychotic activity in specific subgroups of population investigated, such as early-in-onset episodes and no history of atypical antipsychotic drug treatment which is known to epigenetically down-regulate mGlu2 receptors in mice, supporting the hypothesis that the additional preclinical studies and the identification of an appropriate target subgroup with altered glutamatergic tone are required to study these compounds (Kinon et al., 2015).

## The *Epigenetic* “Endophenotypical” Mouse Model for Schizophrenia (PRS): Role of Metabotropic Glutamate 2/3 Receptors

Urged by the need to use a neurodevelopmental animal model to study the epigenetic status at each neurodevelopmental stage of schizophrenia, we investigated the molecular and behavioral abnormalities found in the brain of the offspring of dams stressed during pregnancy (PRS mice). PRS mice showed a marked and long-lasting increase in DNMT enzymes (both 1 and 3a), and TET enzymes and a significant increase in 5-methylcytosine (5MC) and 5-hydroxymethylcytosine (5HMC) in the promoters of putative schizophrenia-related genes, such as *bdnf* , *gad1, reln*, and the early inducible gene, *GADD45* (growth arrest DNA demage), associated with an alteration in these gene expression.

To the best of our knowledge, the PRS model represents a promising model to study the *natural course* of major psychosis including schizophrenia compared, for example, to the *phencyclidine* (PCP) model which is a well-established pharmacological-induced model for schizophrenia reflecting the positive symptoms through the blockade of the NMDA receptors. Schizophrenia and autism spectrum disorders are considered diseases of *neurodevelopment*, characterized by a natural course, starting with a prodromal phase, first episode during adolescence or early adulthood, followed by relapses/remitted periods and eventually leading to brain function deterioration that ensues over subsequent adult years. Hence, the epigenetic history of such complex neurodevelopmental disorders cannot be adequately studied only in the postmortem brains of chronic SZ patients. We then have focused on studying the epigenetic signature of schizophrenia in offspring of PRS mice.

We also used *PRS* mice for the study of the role played by mGlu2 and mGlu3 receptors in the pathophysiology of schizophrenia based on clinical findings with pomeglumetad methionyl, an oral prodrug of the mGlu2/3 receptor agonist, LY404039. This drug showed an efficacy similar to the comparator, olanzapine, on positive and negative symptoms of schizophrenia in a phase-2 clinical trial (Patil et al., 2007), but not in subsequent trials. However, an exploratory analysis of all clinical studies confirmed the antipsychotic activity of pomeglumetad in schizophrenic patients who were early-in-disease or had not been treated with atypical antipsychotic drugs (Kinon et al., 2015).

We found that expression of mGlu2 and mGlu3 receptors was reduced in the frontal cortex of PRS mice (Matrisciano et al., 2012; Holloway et al., 2013), as a result of an increased binding of DNMT1 and methylcytosine binding protein-2 (MeCP2) to the *Grm2* gene promoter (Matrisciano et al., 2012; Figure 1).

As summarized in Table 1, PRS adult offspring showed alterations in the epigenetic regulation of schizophrenia-related gene as reelin, GAD67, BDNF, and mGlu2/3 receptors. Behaviorally, adult PRS-mice showed deficits similar to those observed in psychotic patients such as abnormalities in social interaction, locomotor activity, and pre-pulse inibition (PPI). In addition, we found epigenetic abnormalities such as a marked increase in the expression of DNMT1, DNMT3a, and TET, a significant increase in 5-methylcytosine (5MC) and 5-hydroxymethylcytosine (5HMC) in the promoters of putative schizophrenia-related genes, such as *bdnf* , *gad1, reln*, and the early inducible gene, *GADD45* (growth arrest DNA demage). Interestingly, the biochemical and behavioral abnormalities of PRS mice were corrected by the treatment with LY379268 (Matrisciano et al., 2012; Holloway et al., 2013), an orthosteric agonist of mGlu2/3 receptors, which shows “therapeutic efficacy” in a range of animal models used to predict antipsychotic activity (Cartmell et al., 1999, 2000; Carter et al., 2004). In PRS mice, considered by us as a neurodevelopmental endophenotypical model for schizophrenia, expression levels of epigenetic biomarkers can be assessed at different phases of development in order to further elucidate the underlying pathogenetic mechanisms and predicting treatment responses at specific stages of the disease, with particular attention to early detection and possibly early intervention.

Little is known on the action of antipsychotics on specific epigenetic mechanisms in GABAergic or glutamatergic neurons. Thus, PRS mice represent a valid and suitable model for drug testing and development.

## Clozapine and The mGlu2/3 Receptor Agonist LY379268: Epigenetic Effects in the PRS Mouse Model for Schizophrenia

Clozapine, the prototype of atypical antipsychotics, is considered the drug of choice in patients with treatment-resistant schizophrenia due, in our opinion, to its unique chromatin remodeling properties. We have shown that clozapine reversed the behavioral deficits and induced chromatin remodeling in PRS mice that are resistant to haloperidol treatment (Dong et al., 2016). We recently studied the epigenetic mechanisms underlying the efficacy as potential antipsychotic-like activity of the mGlu2/3 receptors agonist, LY379268, as compared to the activity of clozapine, in PRS mice. Table 2 summarizes the epigenetic effects of clozapine and LY379268 in the frontal cortex of PRS mice. Clozapine reversed promoter hypermethylation of schizophrenia-related genes such as *bdnf* , *reln*, and *gad1* (Dong et al., 2016). Interestingly, these effects were shared by valproate, an anti-epileptic drug used for the treatment of bipolar disorder, which is chemically unrelated to clozapine, and induces demethylation of gene promoters presumably as a result of histone acetylation and chromatin opening. Both clozapine and LY379268 were able to reduce the overexpression of DNMT1 and TET found in the frontal cortex of PRS mice. This overexpression is similar to that found in brain tissue of patients affected by schizophrenia (Matrisciano et al., 2016). DNMT enzymes are responsible for the conversion of cytosines into 5-methyl-cytosines, whereas TET enzymes convert the 5MC residues into 5-hydroxymethylcytosines by hydroxylation reaction in a sequence of events of cytosines metabolism. Clozapine and LY379268 were also able to reverse the hypermethylation of schizophrenia-like promoter genes such as *gad1, bdnf* , and *reln* and the ensuing increase in their mRNA expression levels. In addition, LY379268 induced a decrease of MECP2 binding at the *mGlu2, Gad1*, and *Bdnf* gene promoters, whereas clozapine reversed DNMT binding at the promoters of schizophrenia-related genes. Both clozapine and LY379268 reversed the increase in locomotor activity in PRS mice and the deficits showed by these mice in social interaction tasks. These findings are consistent with the previous evidence that a combined treatment with clozapine and valproate reversed the downregulation of GAD67 expression induced by repeated methionine administration in mice (Guidotti et al., 2014). The same authors showed that the effects of clozapine on DNA-demethylation were mimicked by antipsychotic drugs chemically related to clozapine, such as the dibenzodiazepines, quetiapine and olanzapine, but not by the chemically unrelated risperidone (Guidotti et al., 2011). Thus, a more systematic and comprehensive analysis of the effects of different antipsychotics on the epigenetic signature in PRS mice is warranted. We reported that the strong effect of clozapine on DNA methylation in PRS mice and the lack of effect of clozapine in control mice cannot be considered as secondary to changes in dopaminergic or serotonergic genes such as D2, Htr1a, or Htr2a in the cortex of PRS mice. Of note, a correlation between the methylation state of schizophrenia-related genes and behavioral deficits exists (Dong et al., 2016). The increase in DNMT1 binding to selected *Gad1, Reln*, and *Bdnf-ix* regulatory regions in PRS mice was considerably reduced by clozapine treatment whereas haloperidol failed to reduce the increased DNMT1 binding in PRS mice, in agreement with previous results (Matrisciano et al., 2013). Clozapine and LY379268 may exert their antipsychotic activity either indirectly by decreasing DNMT and TET expression levels, and/or more directly by interfering with the DNMT1 or MeCP2 DNA-binding domains. In addition, both clozapine and LY379268 increased the expression levels of Gadd45-β (growth arrest and DNA-damage-inducible protein 45), a member of the Gadd45 family of small nuclear acidic proteins, which it was reported to facilitate DNA de-methyation (Ma et al., 2009; Matrisciano et al., 2011). Taken together, we can speculate that both clozapine and mGlu2/3 receptor agonists act in our model as epigenetic de-methylating agents, and because of that they may regulate processes that lies at the core of the pathophysiology of schizophrenia.

**Table 2:** Comparison of epigenetic and behavioral abnormalities induced by mGlu2/3 receptors agonist LY379268 and clozapine in PRS mice.

**Clozapine**	**LY379268**
Increase in Gadd45-β expression	Increase in Gadd45-β expression
Reduction of the overexpression of DNMT1 and TET1 in frontal cortex	Reduction of the overexpression of DNMT1 and TET1 in frontal cortex
Reduction of Gad1, Reln, and Bdnf promoter hypermethylation and increase in their mRNA levels	Reduction of the MeCP2 binding at the mGlu2, Gad1, Bdnf gene promoters
Reversal effects of the binding of DNMT1 to unmethylated target promoters	Reversal effects of the binding of MeCP2 to unmethylated target promoters
Reduction in locomotor hyperactivity and deficits in SI	Reduction in locomotor hyperactivity and deficits in SI

*BDNF, brain-derived neurotrophic factor; DNMT1, DNA-methyltransferase 1; TET1, ten-eleven methylcytosine dioxygenase 1. Gadd45 (Growth – arrest and DNA damage; MeCP2, Methylcytosine binding protein 2*.

## Conclusions

This review underlies the concept that the *PRS* mouse model has construct and face validity as an experimental epigenetic model of vulnerability for neurodevelopmental disorders such as schizophrenia, schizoaffective disorders, and autism. This mouse model is highly reproducible and useful for novel anti-psychotic drug screening acting on altered epigenetic mechanisms. Early-life stressors, even during pregnancy, in mice lead to alterations of some molecular players of epigenetic mechanisms that are translated into a schizophrenia-like phenotype. A potential glutamate-based pharmacotherapy for schizophrenia remains, at least in part, a possibility that requires the identification of an appropriate subgroup of patients that satisfy specific criteria such as no previous history of atypical antipsychotic treatments and treatment onset in early phases of the disease. For preclinical studies, PRS mice represent a valid epigenetic “endophenotype” model for drug testing and development and for studying the pathogenesis of the disease. In our opinion, mGlu2/3 receptors, based on the peculiar role as *modulators* of glutamate transmission in the frontal cortex, can still represent a suitable target for novel antipsychotic medications targeting specific high-risk population with dysregulation of brain glutamatergic tone. Then, ligands acting on mGlu2 and 3 receptors, either orthosteric agonists or PAMs, require further experimental studies in PRS mice and other epigenetic models to identify the *optimum* receptors target and time window of intervention in the treatment of psychosis.

## Author Contributions

All authors listed have made a substantial, direct and intellectual contribution to the work, and approved it for publication.

### Conflict of Interest Statement

The authors declare that the research was conducted in the absence of any commercial or financial relationships that could be construed as a potential conflict of interest.
